# Environmentally-Friendly Green Approach for the Production of Zinc Oxide Nanoparticles and Their Anti-Fungal, Ovicidal, and Larvicidal Properties

**DOI:** 10.3390/nano8070500

**Published:** 2018-07-06

**Authors:** Naif Abdullah Al-Dhabi, Mariadhas Valan Arasu

**Affiliations:** Addiriyah Chair for Environmental Studies, Department of Botany and Microbiology, College of Science, King Saud University, P. O. Box 2455, Riyadh 11451, Saudi Arabia; mvalanarasu@ksu.edu.sa

**Keywords:** *Scadoxus multiflorus*, leaf, ZnO NPs, larvicidal, ovicidal, anti-fungal

## Abstract

Green synthesis of nanoparticles can be an important alternative compared to conventional physio-chemical synthesis. We utilized *Scadoxus multiflorus* leaf powder aqueous extract as a capping and stabilizing agent for the synthesis of pure zinc oxide nanoparticles (ZnO NPs). Further, the synthesized ZnO NPs were subjected to various characterization techniques. Transmission electron microscope (TEM) analysis showed an irregular spherical shape, with an average particle size of 31 ± 2 nm. Furthermore, the synthesized ZnO NPs were tested against *Aedes aegypti* larvae and eggs, giving significant LC_50_ value of 34.04 ppm. Ovicidal activity resulted in a higher percentage mortality rate of 96.4 ± 0.24 at 120 ppm with LC_50_ value of 32.73 ppm. Anti-fungal studies were also conducted for ZnO NPs against *Aspergillus niger* and *Aspergillus flavus*, which demonstrated a higher inhibition rate for *Aspergillus flavus* compared to *Aspergillus niger*.

## 1. Introduction

Currently, nanotechnology is a field of intense interest. The process of nanotechnology has been generally classified into three techniques: computational, wet, and dry. While the computational process deals solely with nano-sized structures, the wet process deals with components present in the cells, tissues, and membranes of living organisms. Additionally, the dry process deals with the synthesis of inorganic materials with the help of physical chemistry techniques. The major function of nanotechnology is said to be the synthesis of nanoparticles, mainly relying on the three methodologies such as physical, chemical and biological methods. Of these methodologies, biological synthesis plays a major role when compared with the two other methodologies [1,2,3,4,5]. Biologically-mediated synthesis is further classified into eco-friendly synthesis, which is comprised of plants and plant sources with the corresponding advantages of simplification and lower cost [6,7,8,9,10,11,12,13]. Therefore, we decided to mainly focus on the green synthesis of nanoparticles. For this method of green synthesis of nanoparticles, our research group chose *Scadoxus multiflorus* (*S. multiflorus*) leaf powder aqueous extract (SA) as a green source. This plant is also said to be one of the ancient medicinal plants of India, and belongs to the Caesalpiniaceae family. Different sources of this plant are highly recommended for various treatment purposes, such as irregular menstruation [14]. *S. multiflorus* is a bulbous plant found in most of sub-Saharan Africa which has been used as traditional medicine.

Metal oxide nanoparticles have various significant application possibilities, such as anti-microbial, cell line studies and dye degradation properties. Zinc oxide nanoparticles (ZnO NPs) have a band gap of 3.37 eV, which is relevant for various human applications [15,16].

In this manuscript, we synthesized ZnO NPs with the help of SA. Furthermore, the synthesized ZnO NPs were used to treat one of the major diseases, dengue fever, causing death in India. Dengue is a global disease, with nearly 3 million people affected [17]. *Aedes ageypti* has been stated to be a common vector for causing dengue fever [18]. This manuscript concludes that ZnO NPs are anti-fungal agents effective against *Aspergillus flavus* (*A. flavus*) and *Aspergillus niger* (*A. niger*). Many researchers had reported on the anti-fungal activity of ZnO NPs, which proved to us that ZnO NPs could be utilized as fungicidal agents [19,20,21,22,23].

Overall, this manuscript describes the green synthesis of ZnO NPs using SA, and the subjection of the synthesized particles to various application studies, such as larvicidal and ovicidal activities against *Aedes ageypti* (*A. ageypti*). Furthermore, the synthesized particles, subjected to two different fungal strains, i.e., *A. flavus* and *A. niger*, were studied and are reported herein.

## 2. Materials and Methods

### 2.1. Materials and Reagents

The *S. multiflorus* leaf powder was directly procured from the local market and utilized in our research. Zinc acetate was obtained from Sigma-Aldrich (Riyadh, Saudi-Arabia). Reverse-osmosis and double-distilled water was used for the other experiments performed in this study.

### 2.2. Extraction of the Scadoxus multiflorus Leaf Powder Aqueous Extract Sample

The 30 g of procured powder material of the *S. multiflorus* leaf was immersed in 100 mL of distilled water and placed in a water bath at 60 °C for 1 h. Then, the solvent and powder layer were separated using a Buchner funnel and Whatmann filter paper. The filtrate solution of SA was collected and stored in a refrigerator to be utilized for the future synthesis of ZnO NPs.

### 2.3. Production of Zinc Oxide Nanoparticles

By using a pipette and mechanical stirrer, 20 mL of collected SA filtrate was added, drop by drop, to 80 mL of 1 mM of zinc acetate under stirring at room temperature (RT). Then, the resultant solutions were placed in a water bath at 60 °C for 3 h and monitored using UV–visible spectroscopy (Hitachi, Tokyo, Japan). Once the reaction mixtures confirmed the formation of ZnO NPs, the resultant solution was subjected to centrifugation at 3000 rpm for 20 min. The centrifugation processes were repeated three times with the help of distilled water to synthesize pure ZnO NPs. Once the centrifugation process was over, the supernatant was discarded, and the pellets were collected and placed in a furnace at 400 °C to obtain the desired product in powder form.

### 2.4. Analytical Techniques

After synthesis of the ZnO NPs, various analytical techniques, such as UV–visible spectrophotometry (Hitachi, Tokyo, Japan) were performed for the determination of the absorption maximum of the particles. The prepared material was mixed along with KBr to form pellets, to determine the Fourier-transform infrared (FTIR) spectroscopy, using a Shimadzu FTIR Spectrophotometer (Hitachi, Tokyo, Japan). The crystalline nature of the material was characterized by applying an X-ray diffractometer (XRD) (Model D8, Bruker, Germany). Transmission electron microscopy (TEM) (FEI company, Hillsboro, OR, USA) was performed to determine the morphology of the material. A particle-size histogram was developed using Image J software and the Zeta potential was determined by a Horiba nanoparticle analyzer (Horiba scientific, Kyoto, Japan), to identify the stability of the nanoparticles. Shimadzu atomic absorption spectrometry (Shimadzu, Kyoto, Japan) was used to determine Zn, with the help of a deuterium lamp.

### 2.5. Larvicidal and Ovicidal Properties of Synthesized Zinc Oxide Nanoparticles

*Aedes ageypti* (*A. ageypti*) larvae were cultured in the laboratory at RT. The third instar larvae were collected and utilized for larvicidal studies; the eggs were collected for ovicidal activity under various concentrations of ZnO NPs—15, 30, 60, and 120 ppm—which were studied and reported using a MANOVA; LSD-DMRT Test. LC50 and LC90 values were also calculated, and identified to be statistically significant at *p* < 0.05. In this study, Neem azal, which is a commercially available insecticide, was utilized as the standard for ovicidal activity [24,25].

### 2.6. Antifungal Activity of Zinc Oxide Nanoparticles 

The studied fungal strains, such as *A. flavus* MTCC 873 and *A. niger* MTCC 282, were procured from IMTECH (Chandigarh, India) and were then processed by using Clinical Laboratory and Standard Institute (CLSI) methods. An amount of 100 mL of PDB (Potato Dextrose Broth) was autoclaved, and *A. flavus* and *A. niger* fungal strains were inoculated into the broth. Test samples of 1 mg/mL were placed in an incubator while being stirred at 120 rpm at RT. After two weeks, the strains were collected, and the biomass of the fungi was filtered and kept for drying. This dried biomass was utilized for further studies, with carbendazim as the standard [26,27]. The mortality percentage of the fungal biomass was calculated using the formula below.

$$\frac{\mathrm{Weight}\;\mathrm{of}\;\mathrm{the}\;\mathrm{control}-\mathrm{Weight}\;\mathrm{of}\;\mathrm{the}\;\mathrm{test}\;}{\mathrm{Weight}\;\mathrm{of}\;\mathrm{the}\;\mathrm{control}}\;\times \;100$$

## 3. Results and Discussion

### 3.1. UV–Visible Spectroscopy

**Figure 1:**
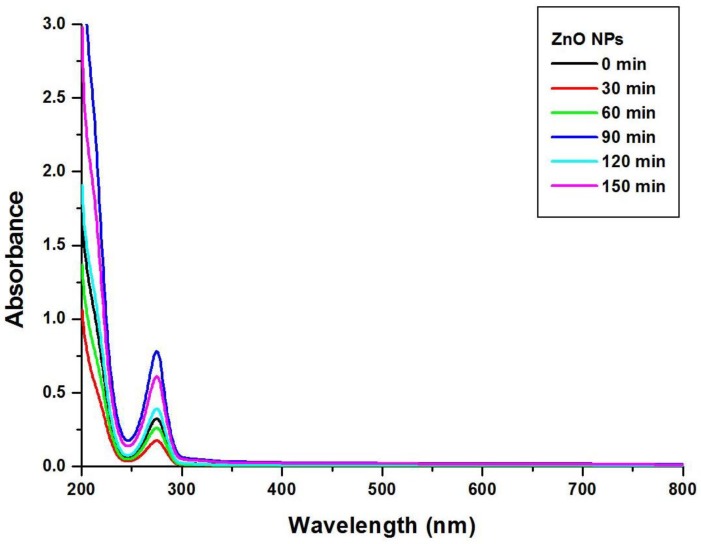
UV–visible spectroscopy of ZnO NPs.

The reaction mixtures of SA and zinc acetate were monitored using UV–visible spectroscopy at the wavelengths of 200 to 800 nm. From the observed results it can be inferred that the highest absorbance of 274 nm is at 90 min, which relies on the conversion of the starting material to end product, as clearly illustrated in Figure 1.

### 3.2. FTIR Analysis of Zinc Oxide Nanoparticles

**Figure 2:**
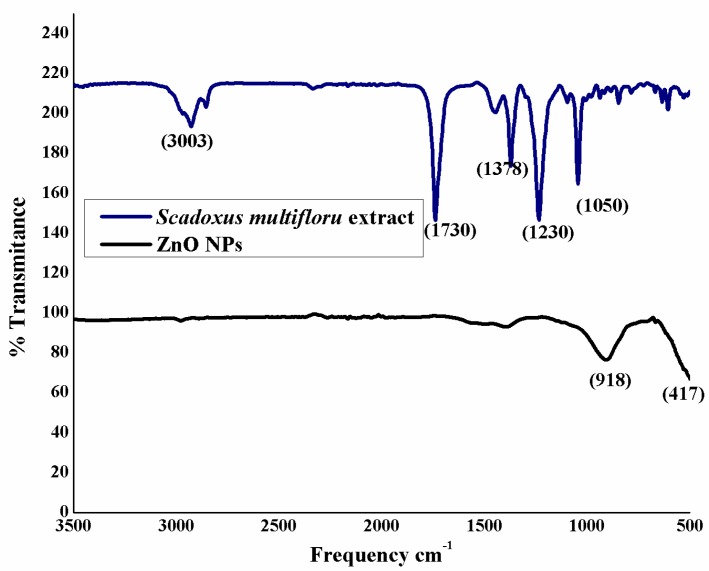
FTIR analysis of ZnO nanoparticles and extract.

The specimens were subjected to FTIR study, as illustrated in Figure 2. Sample *S. multiflorus* leaf extract and ZnO NPs were both recorded to give the FTIR spectra. The FTIR spectrum of the *S. multiflorus* leaf extract shows peaks at 3003 and 1730 cm^−1^, which correspond to functional groups such as –C=O and C–H (stretch), present in organic molecules. These peaks completely disappear in the ZnO NPs spectrum, which clearly illustrates that the organic molecules are acting as capping and stabilizing agents. The ZnO NPs spectrum showed a characteristic Zn–O stretching at ~417 cm^–1^, which confirms the formation of ZnO.

### 3.3. XRD Analysis of Zinc Oxide Nanoparticles

**Figure 3:**
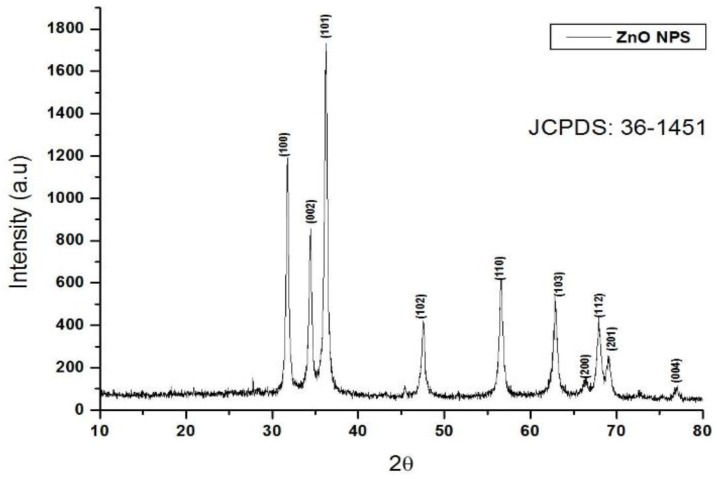
X-ray diffraction (XRD) analysis of the ZnO NPs.

The obtained ZnO NPs were investigated to study their crystalline nature by XRD spectroscopy. From the results it can be inferred that the synthesized ZnO NPs were synthesized in their pure phase, without any impurities. The results also confirmed the h k l values of the (100), (002), (101), (102), (110), (103), (200), (112), (201), and (004) crystalline pattern. Furthermore, the crystalline structure was matched with the JCPDS data of 36-1451, and with the help θ of full-width and half-maximum data, with *d* = 1.64056 and 2θ = 37. Twenty-three plane crystalline data were calculated by Scherrer’s formula *D* = kλ/β cosθ [28]. The synthesized crystalline particles were said to be 31.8 nm in size, as illustrated in Figure 3.

### 3.4. Zinc Oxide Nanoparticles Morphological Studies

**Figure 4:**
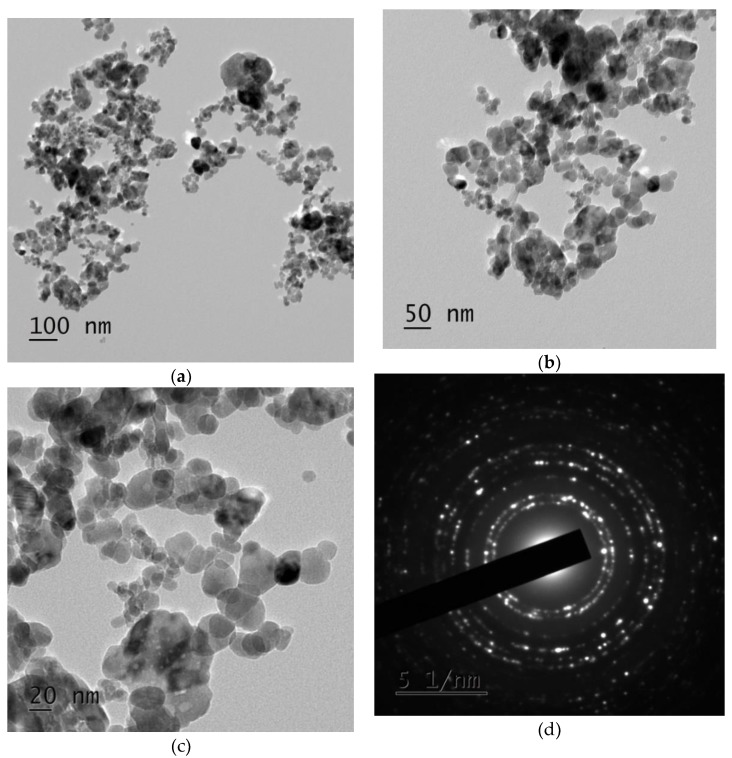

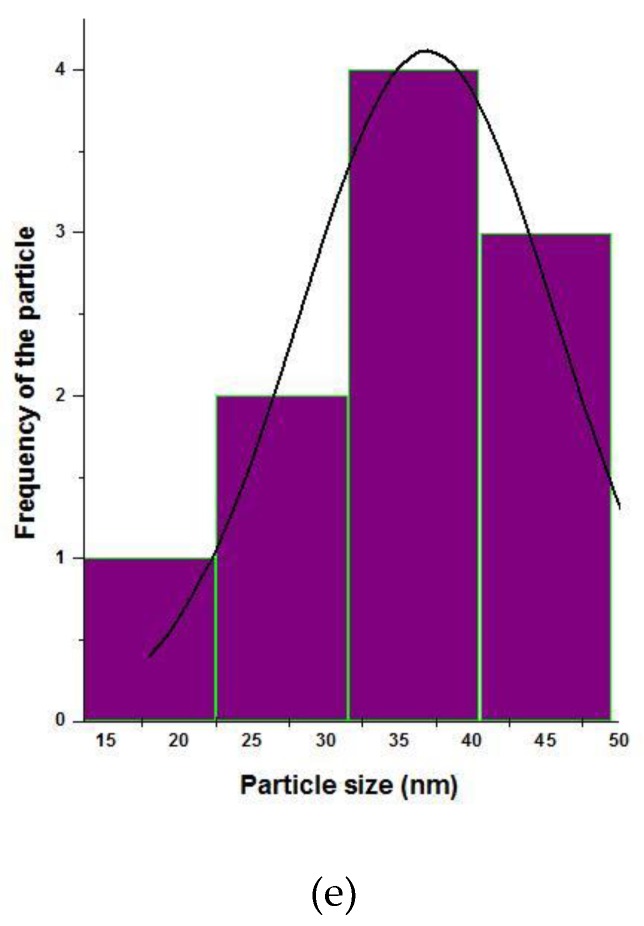
(**a**–**c**) TEM images of ZnO NPs and (**d**) SAED pattern of ZnO NPs-particle size histogram (**e**) Particle size histogram.

Eco-friendly synthesized ZnO NPs were identified by their morphology using transmission electron microscopy (TEM). From the observed results it can be inferred that the synthesized pure ZnO NPs show irregular, spherical-shaped particles, as illustrated in Figure 4a–c. The particles seem to be legitimately agglomerated, with sizes in the range of ~100 nm. The Selected area (electron) diffraction pattern also clearly cuts the crystalline nature of eco-friendly synthesized ZnO NPs, as shown in Figure 4d. This is a typical phenomenon, taking place due to interaction of H_2_O and ZnO. Due to inter-particle interactions, such as van der Waals and electrostatic or magnetic forces, the ZnO NPs in aqueous medium have a tendency to exhibit as an aggregated particle, leading to the development of soft agglomerates. Conversely, particle agglomeration is not complex, because the application purpose (i.e., larvicidal, ovicidal, and fungicidal activity) of the ZnO NPs depends upon the particle size and not on the agglomerate size.

### 3.5. Particle Size Histogram Analysis of the Zinc Oxide Nanoparticles

Our research group utilized the ImageJ software for plotting the particle size histogram. The results show that the eco-friendly synthesized ZnO NPs had an average particle size of 31 ± 2 nm, as shown in Figure 4e.

### 3.6. Energy Dispersive X-ray Analysis (EDAX) Spectrum of Zinc Oxide Nanoparticles

**Figure 5:**
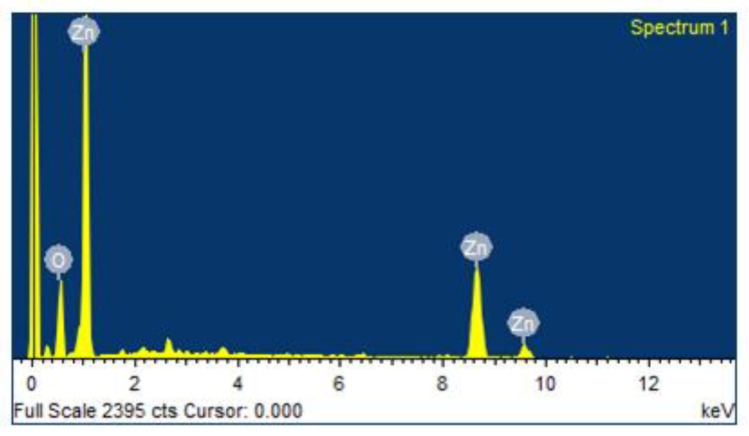
EDAX spectrum of ZnO NPs.

The synthesized ZnO NPs were subjected to an EDAX spectrum to quantify the mixture of metal and oxides present in the sample. The results showed that 64.12% of Zn and 35.76% of O were present on the surface area, as clearly shown in Figure 5.

### 3.7. Stability of Synthesized Zinc Oxide Nanoparticles

**Figure 6:**
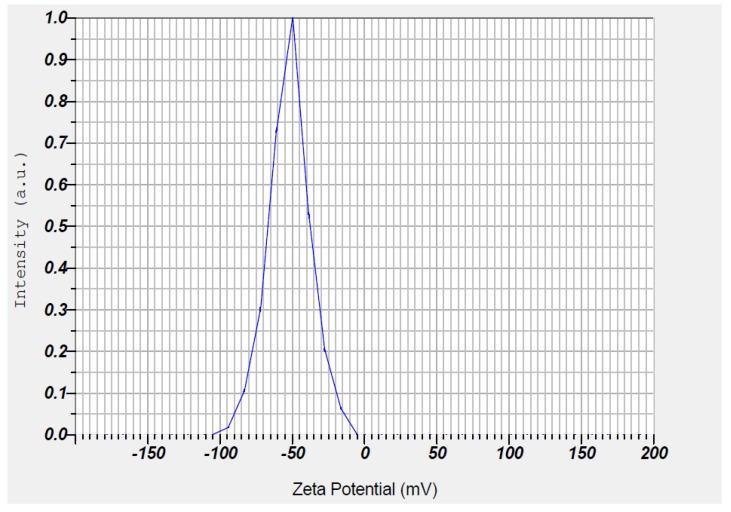
Zeta potential analysis of ZnO NPs.

The resultant ZnO NPs were subjected to determine the Zeta potential to test their stability, which resulted in a value of −51.8 mV, as clearly illustrated in Figure 6.

### 3.8. Atomic Absorption Spectroscopy

**Figure 7:**
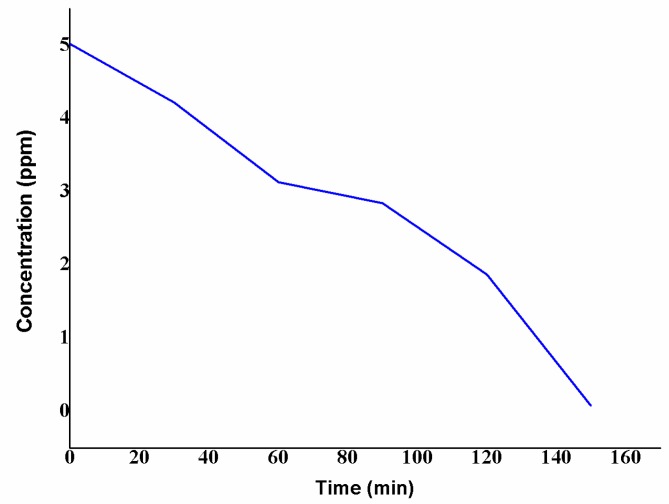
Atomic absorption spectroscopy analysis of zinc acetate in the nanoparticle-forming solution.

The synthesized quantity of ZnO NPs was analyzed by atomic absorption spectroscopy (AAS) after adding the zinc acetate, with the intention of realizing the remaining concentration of zinc. AAS analysis for the nanoparticle preparing solution, performed at regular intervals of time, exhibited the formation of ZnO NPs. Initially, the standard solution of 5.02 ppm of zinc acetate was prepared and analyzed with AAS at 0 min. After adding *S. multiflorus* leaf extract and the zinc acetate, the formation of nanoparticles was observed at regular time intervals (Figure 7). The result showed a decrease in the concentration of zinc (5.02, 4.22, 3.13, 2.84, 1.87, and 0.08 ppm at 30 min intervals, respectively), indicating the conversion of zinc acetate to ZnO NPs. Additionally, in this present study, 1 gram dry weight of *S. multiflorus* leaves could synthesize 1.15 mg of ZnO NPs within 90 min. Furthermore, this is a sustainable method that does not use toxic chemicals.

### 3.9. Larvicidal Activity of Zinc Oxide Nanoparticles

Dengue-causing vectors were treated with ZnO NPs at various concentrations: 15, 30, 60, and 120 ppm. The percentage mortality figures are 1.6 ± 0.4, 28.6 ± 7.5, 42.4 ± 2.5, 82.2 ± 6.4, and 98.4 ± 2.3, respectively. This mortality percentage indicates a dose-dependent reaction at higher concentrations, as well as an increasing death rate. Lastly, with the help of LSD tests, we calculated LC_50_ and LC_90_ values with upper and lower confidence limits, as clearly illustrated in Table 1 showing significant results at *p* < 0.05 [10,28]. When compared to the literature [29], our methodologically synthesized ZnO NPs had less larvicidal activity, which may be due to the absence of a bio-organic phase on the surface of the ZnO nanoparticles. *Sargassum wightii*-mediated prepared ZnO NPs have a higher LC_50_ value (49.22 ppm) compared to our result [30]. In another paper [31], *Ulva lactuca*-fabricated ZnO NPs were screened for larvicidal activity against *A. aegypti*, which showed an IC_50_ value of 22.38 ppm. Our methodology provides highly crystalline, pure, and no-bio-organic-phase ZnO NPs. For the control experiment, 1.6% mortality was recorded. The LC_50_ value for larval toxicity was 34.04 ppm.

### 3.10. Ovicidal Activity of Zinc Oxide Nanoparticles

The eco-friendly synthesized pure form of ZnO NPs was subjected to *A. ageypti* eggs with Neem azal as a standard, with various concentrations: 15, 30, 60, and 120 ppm. The obtained results showed that the ovicidal activity relied on a dose-dependent reaction, with a higher mortality percentage of 96.4 ± 0.24 at 120 ppm. The obtained results after five replicates are depicted in Table 2 [10,28]. Our results relate to the literature [32], i.e., *Terminalia chebula* extracts against *A. ageypti*. The ovicidal activity of ZnO NPs was reported, and may be affected by diverse factors, predominantly egg age and contact period. The egg age influenced the ovicidal action of ZnO NPs. The exposure of freshly laid eggs to ZnO NPs causes higher mortality rates. Our output shows 96.4% mortality at 120 ppm, while *Terminalia chebula* (*T. chebula*) extracts exhibit only 66% mortality. The LC_50_ value for ovicidal toxicity was 32.73 ppm.

ZnO NPs were screened for ovicidal activity against which showed an IC_50_ value of 32.73 ppm. Concerning the mechanisms of action of nanoparticles, Volker et al. noted that nanoparticles can affect various physiological parameters in treated organisms, both in vitro and in vivo. The results of in vitro assays showed dose-dependent cell death with oxidative stress as the main likely toxicity pathway. In addition, silver nanoparticles may affect cellular enzymes by interference with free thiol groups and mimicry of endogenous ions. The nanoparticles affect the physiological process of the target organism [33]. On the other hand, strictly limited specific studies have been carried out to elucidate the precise mechanisms of the action of metal nanoparticles on insect pests and vectors [24,25]. However, in the present study, effort has been made to find the mechanism behind the mortality of the mosquitos. The scientific findings have been claimed that, the death of the mosquito may be due the absorption of the nanoparticle into the system and might affect the epithelial cell/ midgut or cortex [25]. It has been predicted that, when the ZnO nanoparticles were absorbed they gets accumulated in the midgut which leads to the shrinkage abdomen leads to the alteration of the mosquitos system. Alternatively, ZnO may affect the functions of other parts such as thorax and midgut, as well as other effects namely lateral hair loss, deformation in gills as well as brushes. Due to these damages in the system it might be the fact the mosquitoes could not undergo respiration hence forth leads to death.

### 3.11. Zinc Oxide Nanoparticles as Fungicides

**Figure 8:**
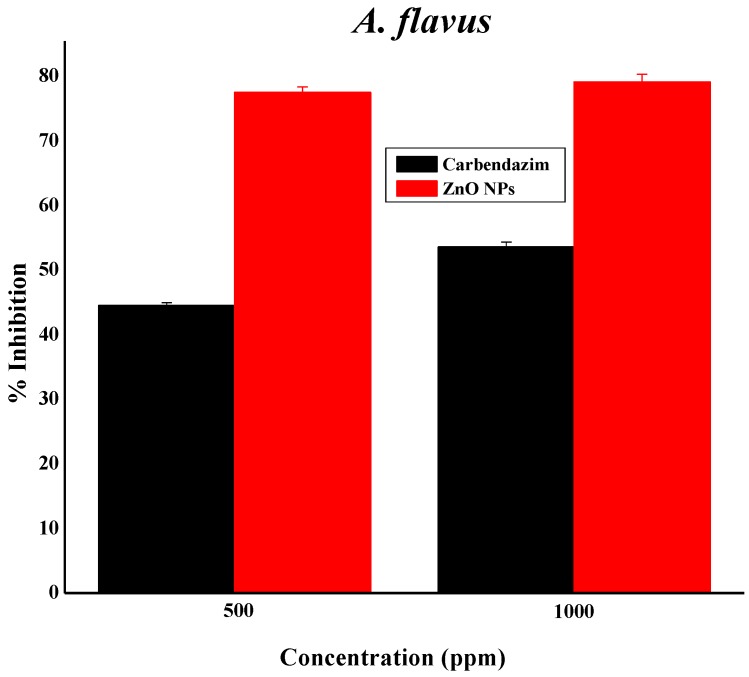

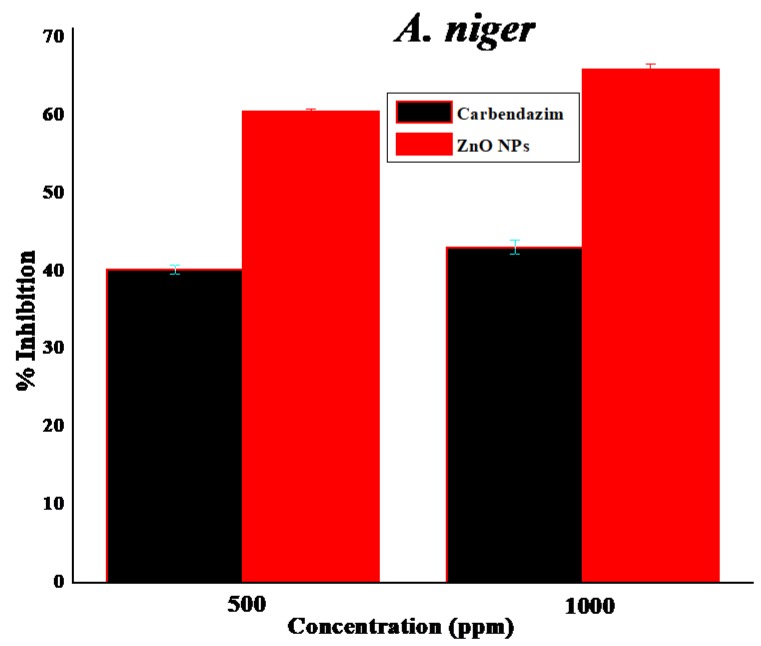
ZnO NPs’ anti-fungal activity against *A. flavus* and *A. niger*.

**Figure 9:**
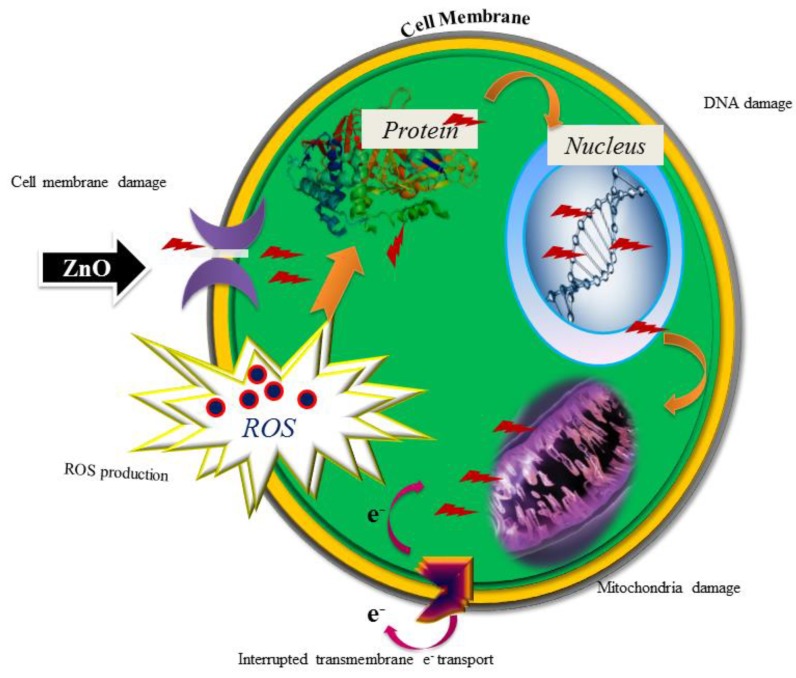
Mode of action of ZnO NPs on microbes.

Green synthesized pure ZnO NPs were subjected to two fungal pathogens: *A. flavus* MTCC 873 and *A. niger* MTCC 282. The ZnO NPs played a prominent role against *A. flavus*, with 75% inhibition at 500 ppm and 76% inhibition at 1000 ppm, while *A. niger* resulted in 57% and 63% inhibition, respectively [27], as clearly illustrated in Figure 8. The results were compared with the reported work [34]. The prepared ZnO NPs are active only at higher concentrations. Therefore, there is not much activity against *A. flavus* and *A. niger*. Many reports are available on ZnO NPs and their biological activity. These reports clearly state that smaller-sized nanoparticles (NPs) will have higher activity [35]. ZnO NPs might be toxic to some strains, but they are considered essential nutrients. The second reason for the antibacterial activity is that when the Zn^2+^ released by ZnO comes into contact with the cell membranes of the microbe, the cell membranes with negative charge and Zn^2+^ with positive charge mutually attract, and the Zn^2+^ penetrates into the cell membrane and reacts with sulfhydryl groups inside the cell membrane. As a result, the activity of synthetase in the microbe becomes so damaged that the cells lose the ability of growth through cell division, which leads to the death of the microbe (Figure 9) [36,37].

## 4. Conclusions

In this manuscript, we proposed a simple process of ZnO NP synthesis by using SA. The results on the synthesized ZnO NPs showed they were irregular, spherical in shape, with an average particle size of 31 ± 2 nm. In addition, the material portrayed promising activity for dengue fever treatment by acting against *A. ageypti*, showing a significant difference at *p* < 0.05. Ovicidal activity was dose-dependent, with an increasing mortality rate at 120 ppm. The activity of the nanoparticles against two fungal pathogens resulted in a higher inhibition rate of *A. flavus* compared to *A. niger*. Moreover, they exhibited effective larvicidal properties against tested fungi and insects. Hence, this study concludes that *S. multiflorus* mediated ZnO NPs may be used as effective control tools against mosquito larval populations and have potential applications in the pharmaceutical and biomedical field.

## Figures and Tables

**Table 1:** Larvicidal activity of synthesized ZnO NPs.

Concentration (ppm)	Mortality * (%)	LC_50_ (ppm)	95% Confidence Limits (ppm)		LC_90_ (ppm)	95% Confidence Limits (ppm)		χ^2^ Value
			LCL	UCL		LCL	UCL	
Control	1.6 ± 0.4 ^a^	34.04	14.82	50.32	78.06	58.75	143.75	3.189
15	28.6 ± 7.5 ^b^							
30	42..4 ± 2.5 ^c^							
60	82.2 ± 6.4 ^d^							
120	98.4 ± 2.3 ^e^							

The value represents the mean ± S.D. of five replications. * mortality of the larvae observed after 24 h of the exposure period, WHO (2005). LC_50_: lethal concentration that causes 50% mortality; LC_90_: lethal concentration that causes 90% mortality. LCL: lower confidence limit; UCL: upper confidence limit. Values in a column with a different superscript alphabet are significantly different at *p* < 0.05 (MANOVA; LSD-DMRT Test).

**Table 2:** Ovicidal activity by green synthesized ZnO NPs.

Concentrations (ppm)	% of Mortality
15	35.5 ± 0.23
30	47.2 ± 1.21
60	63.7 ± 0.38
120	96.4 ± 0.24
Neem azal (120)	100 ± 0.00

Values represent mean ± S.D. of five replications. Different alphabets in the column are statistically significant at *p* < 0.05. (MANOVA; LSD-DMRT Test). Eggs in the control groups were not sprayed with phytochemicals.LC_50_—32.73 ppm; LCL—24.20 ppm; UCL—44.27 ppm.
